# Central Blood Pressure Monitoring via a Standard Automatic Arm Cuff

**DOI:** 10.1038/s41598-017-14844-5

**Published:** 2017-10-31

**Authors:** Keerthana Natarajan, Hao-Min Cheng, Jiankun Liu, Mingwu Gao, Shih-Hsien Sung, Chen-Huan Chen, Jin-Oh Hahn, Ramakrishna Mukkamala

**Affiliations:** 1 Department of Electrical and Computer Engineering, Michigan State University, East Lansing, Michigan, USA; 2 Department of Medicine, National Yang-Ming University, Taipei City, Taiwan; 3 Department of Mechanical Engineering, University of Maryland, College Park, MD, USA

## Abstract

Current oscillometric devices for monitoring central blood pressure (BP) maintain the cuff pressure at a constant level to acquire a pulse volume plethysmography (PVP) waveform and calibrate it to brachial BP levels estimated with population average methods. A physiologic method was developed to further advance central BP measurement. A patient-specific method was applied to estimate brachial BP levels from a cuff pressure waveform obtained during conventional deflation via a nonlinear arterial compliance model. A physiologically-inspired method was then employed to extract the PVP waveform from the same waveform via ensemble averaging and calibrate it to the brachial BP levels. A method based on a wave reflection model was thereafter employed to define a variable transfer function, which was applied to the calibrated waveform to derive central BP. This method was evaluated against invasive central BP measurements from patients. The method yielded central systolic, diastolic, and pulse pressure bias and precision errors of −0.6 to 2.6 and 6.8 to 9.0 mmHg. The conventional oscillometric method produced similar bias errors but precision errors of 8.2 to 12.5 mmHg (p ≤ 0.01). The new method can derive central BP more reliably than some current non-invasive devices and in the same way as traditional cuff BP.

## Introduction

Tonometric devices for non-invasive monitoring of central blood pressure (BP) have been available for many years now. These devices either acquire a carotid artery tonometry waveform and calibrate it with brachial cuff BP levels for a “direct” measurement of central BP or obtain a similarly calibrated, but easier-to-measure, radial artery tonometry waveform and then apply a generalized transfer function (GTF) to the peripheral BP waveform for an indirect measurement of central BP. The devices have even been shown to provide added clinical value over traditional brachial cuff BP measurements in several research studies^1^. Yet, because applanation tonometry of any artery is nontrivial, they have not reached patient care.

**Figure 1:**
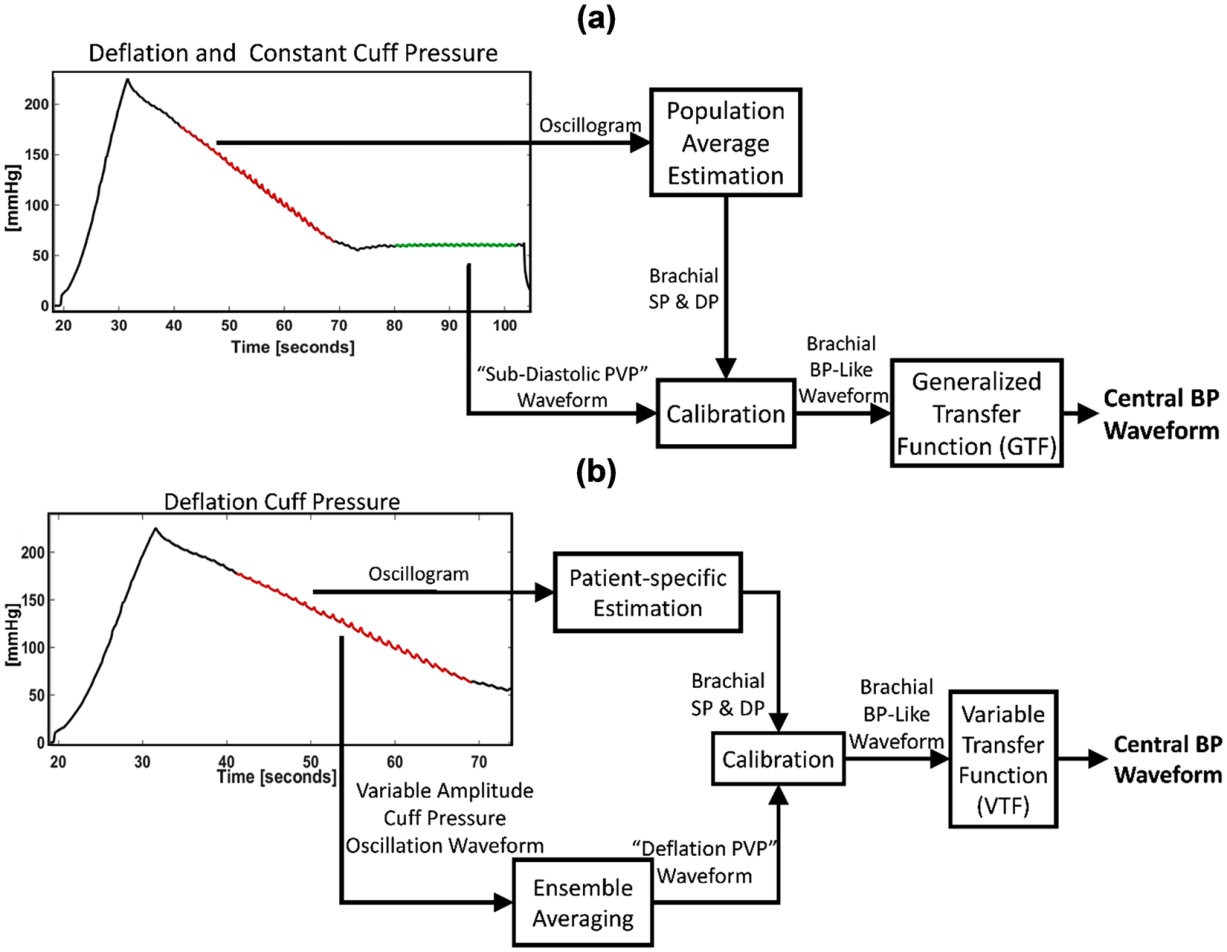
(**a**) Conventional method for monitoring central blood pressure (BP) via a special automatic arm cuff device. The oscillogram is the variable cuff pressure oscillation amplitude versus cuff pressure function; PVP, pulse volume plethysmography; SP, systolic BP; DP, diastolic BP. **(b)** Physiologic method for monitoring central BP via a standard automatic arm cuff device. The sub-methods are shown in Fig. 2.

As a result, oscillometric devices for more convenient monitoring of central BP have recently been introduced^2,3^. As indicated in Fig. 1a, these devices employ a special automatic arm cuff to derive central BP generally in four steps. First, brachial BP levels are obtained in the standard way by slowly deflating (or inflating) the cuff and then estimating the values from the oscillogram (i.e., the variable cuff pressure oscillation amplitude versus cuff pressure function). Second, a fixed amplitude cuff pressure oscillation or “pulse volume plethysmography (PVP)” waveform is measured by maintaining a constant cuff pressure around the diastolic level for up to 30 sec^4–11^ or even above the systolic level by up to 35 mmHg^12,13^. Third, a brachial BP-like waveform is derived by calibrating the PVP waveform with the brachial BP levels. Fourth and finally, central BP is computed from the peripheral waveform typically via a GTF. The error in the measured central BP can be substantial^2^. Like the tonometric devices, the main error source is the error in the brachial BP levels used for calibration^2,14,15^. This latter error can be large, because automatic arm cuffs employ population average methods to estimate the brachial BP levels^16–18^. A secondary error source may be error arising from the use of a one-size-fits-all GTF.

Our broad objective is to achieve accurate central BP monitoring via a standard automatic arm cuff. In recent studies, we developed a patient-specific method for estimating brachial BP levels from the oscillogram by leveraging physiologic modeling and optimization^19^ and showed that this method can be more accurate in estimating the brachial BP levels than widely used population average methods^20^. We also previously proposed adaptive transfer functions likewise based on modeling and optimization to derive the central BP waveform from a directly measured peripheral BP waveform^21,22^. In this study, our new contributions were to (1) conceive simple, yet physiologic, methods to (a) extract the PVP waveform from the variable amplitude cuff pressure oscillation waveform and (b) vary the transfer function relating calibrated PVP to central BP with BP-induced changes in pulse transit time; (2) integrate these methods with the patient-specific method to calibrate the PVP waveform and thereby derive central BP; and (3) assess the integrated, “physiologic” method against a high fidelity aortic catheter in a challenging set of patients. Our results indicate that the physiologic method can derive central BP more reliably than some current non-invasive devices and in the exact same way as traditional brachial cuff BP.

## Materials and Methods

### Physiologic Method for Central BP Monitoring via a Standard Automatic Arm Cuff

The developed method is based on physiologic modeling and knowledge and is overviewed in Fig. 1b. This physiologic method computes the central BP waveform from a cuff pressure waveform obtained only during conventional deflation (or inflation) via successive application of three sub-methods as follows. First, a patient-specific method is applied to an oscillogram (derived from the waveform) to yield brachial systolic and diastolic BP (SP and DP). Then, an ensemble averaging/calibration method is applied to the variable amplitude cuff pressure oscillation waveform (obtained by high pass filtering the cuff pressure waveform) to extract a “deflation PVP” waveform and scale it to the brachial BP levels. Finally, a variable transfer function (VTF) method is employed to convert the brachial BP-like waveform to the central BP waveform. Each of these three sub-methods is further described below.

### Patient-Specific Method for Estimation of Brachial SP and DP

**Figure 2:**
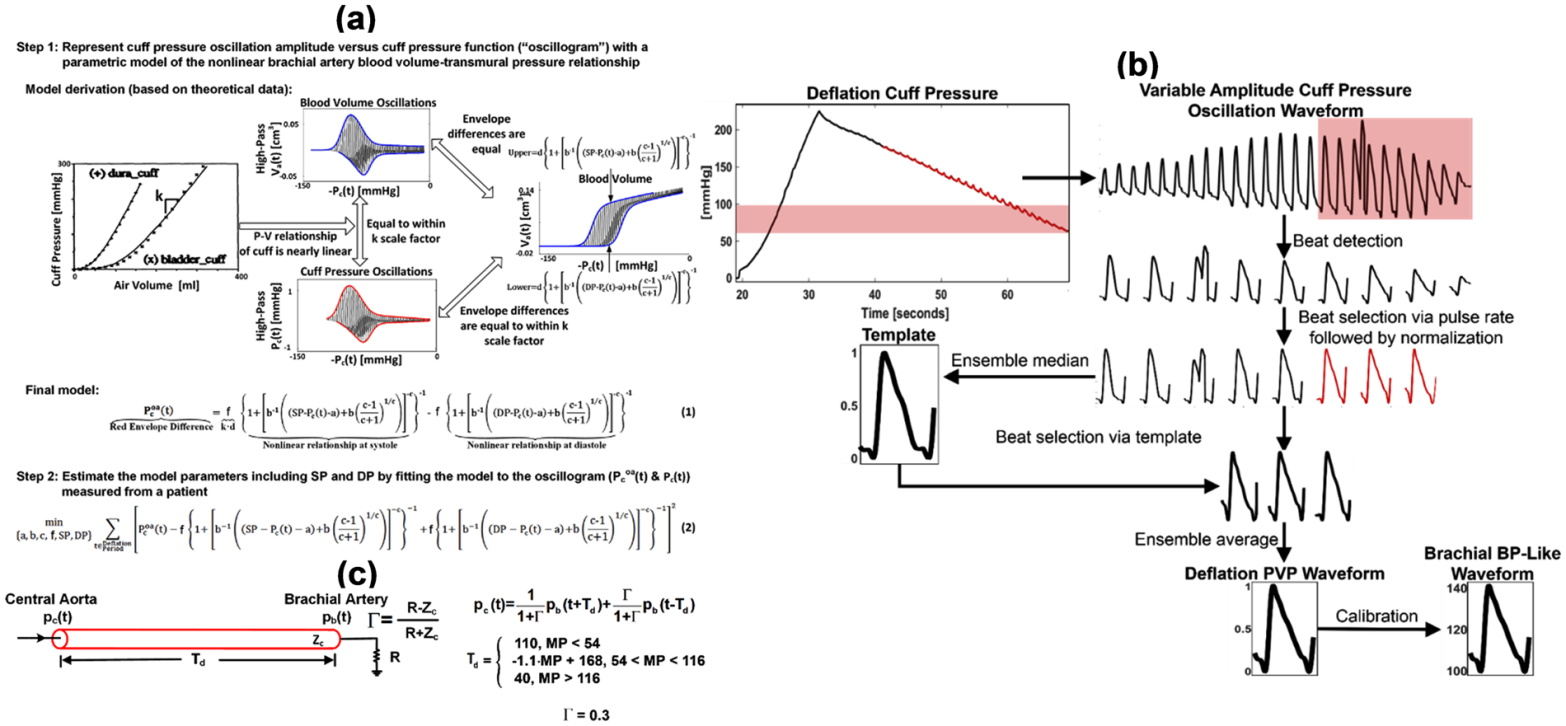
(**a**) Patient-specific method for estimating brachial BP levels from a cuff pressure waveform obtained during conventional deflation by leveraging a physiologic model and optimization. The method is described in detail in^19^; and figure adapted from^20^ © 2016 IEEE. Reprinted, with permission, from IEEE (IEEE Transactions on Biomedical Engineering). **(b)** Ensemble averaging/calibration method for extracting a brachial BP-like waveform from the cuff pressure waveform obtained during conventional deflation. The method extracts a deflation PVP waveform by normalizing and then averaging similar waveform beats from the deflation end (wherein PVP and BP waveform shapes may better agree due to relatively constant brachial artery compliance) and then scales the waveform to brachial SP and DP. **(c)** Variable transfer function (VTF) method for converting the brachial-like BP waveform to the central BP waveform. The method defines the transfer function in terms of the pulse transit time (T_d_) and wave reflection coefficient (Γ) parameters of an arterial tube-load model and then varies T_d_ based on its well-known inverse relationship with mean BP (MP). MP is estimated as the time average of the brachial BP-like waveform. The model parameter values were defined via the training dataset (see Table 1). T_d_ is in units of msec; MP, mmHg.

The patient-specific method is shown in Fig. 2a and described in detail elsewhere^19^. Briefly, as explained in^19,20^, the oscillogram (difference between the upper and lower envelopes in red in Fig. 2a) is represented with a physiologic model accounting for the nonlinear brachial artery blood volume-transmural pressure relationship (see Eq. (1) in Fig. 2a). In particular, the nonlinear relationship is represented with a sigmoidal function as justified by experimental data^23^, and the model of the oscillogram is then specified as the nonlinear relationship evaluated at brachial SP (see upper blue envelope in right plot of Fig. 2a) minus the nonlinear relationship evaluated at brachial DP (see lower blue envelope in same plot). This model arises from two observations. First, the difference in the upper and lower envelopes of the blood volume waveform as a function of negative cuff pressure is essentially equivalent to the difference in the upper and lower envelopes of the blood volume oscillations (i.e., the high pass filtered blood volume waveform) as a function of negative cuff pressure (compare right and upper plots in Fig. 2a). Second, the cuff pressure-air volume relationship of actual cuffs is nearly linear over a wide range (see left plot in Fig. 2a). Hence, the unmeasured blood volume oscillations may be proportional to the measured cuff pressure oscillations (compare upper and lower plots in Fig. 2a) with a proportionality constant equal to k, which indicates the reciprocal of the compliance of the cuff. The model parameters therefore represent brachial SP and DP and brachial artery mechanics [a, b, c, f]. In terms of the brachial artery compliance curve (derivative of the nonlinear relationship with respect to transmural pressure), a denotes the transmural pressure at which the curve is maximal; b and c indicate the width of the curve and extent of asymmetry about its maximum; and f equals the scale factor for the curve [d] times k. As buttressed by directly measured compliance curves^23^, a is fixed so that the curve peaks near zero transmural pressure, and b is constrained by the value of c such that the curve is right-skewed. The remaining four patient-specific parameters (i.e., brachial SP, brachial DP, c, f) are then estimated by constrained least squares fitting of the model to the oscillogram (see Eq. (2) in Fig. 2a). The user-selected variables (most notably, the a and b constraints) were established using a training dataset comprising cuff pressure waveforms for analysis and invasive reference brachial BP waveforms from cardiac catheterization patients (see Patient Data). Note that this method effectively employs a variable equation to estimate the brachial BP levels from the oscillogram. The variables in the equation represent the brachial artery compliance curve but do not indicate the placement of the cuff. Hence, the method is specific to the patient in terms of the former but not the latter.

### Ensemble Averaging/Calibration Method for Estimation of the Brachial BP Waveform

The patient-specific method also outputs the entire brachial BP waveform via additional steps dictated by its underlying model. While this waveform is suitable for estimating mean BP (MP), it contains some artifact caused by inter-beat cuff pressure variations. Hence, another method is applied to extract a brachial BP-like waveform from the variable amplitude cuff pressure oscillation waveform. This method is simpler but still founded in physiology. In particular, each beat of the waveform not only varies in amplitude but also in shape. The shape variations are likewise due (in part) to the brachial artery compliance changes with transmural pressure^23^. Since this compliance may be relatively constant over the higher transmural pressure range of oscillometry (e.g., 50 mmHg) wherein elastin fibers play a greater role in arterial wall mechanics^24^, the shape of a beat of the waveform may better reflect that of the brachial BP waveform at lower cuff pressures (e.g., 50 mmHg). Hence, a deflation PVP waveform is extracted from the variable amplitude waveform over the lower cuff pressure range via robust ensemble averaging and calibrated to the brachial BP levels.

The ensemble averaging/calibration method is shown in Fig. 2b. The variable amplitude cuff pressure oscillation waveform is analyzed over the cuff pressure range extending from (i) the minimum cuff pressure analyzed by the patient-specific method minus 40 mmHg to (ii) the minimum cuff pressure analyzed by the patient-specific method (red shading). The waveform beats are detected. To eliminate anomalies, all waveform beats of lengths within 30% of the average beat length are selected. If fewer than three waveform beats meet this criterion, then the three waveform beats with lengths closest to the average beat length are selected. Each selected waveform beat, including 250 msec intervals before the first foot and after the last foot, is equalized by normalization to peak amplitude of one and feet amplitudes of zero. (Time normalization could also be employed, if necessary, to further equalize the waveform beats.) To further eliminate anomalies, a template waveform beat is constructed by computing the ensemble median of all selected waveform beats over the minimum beat length and then applying the same normalization. The three waveform beats with root-mean-squared-error (RMSE) < 0.5 relative to the template waveform beat that are nearest to the minimum cuff pressure are selected (red traces). If less than three waveform beats meet this criterion, then the three waveform beats with the lowest RMSEs are selected. The ensemble average of the selected waveforms beats is computed over the minimum beat length and likewise normalized to yield the deflation PVP waveform. This waveform is then scaled to brachial SP and DP to yield a brachial BP-like waveform. All user-selected variables (e.g., 30% beat length and 0.5 RMSE thresholds) were defined with a training dataset (see Data Analysis).

### VTF Method for Estimation of the Central BP Waveform

The VTF method is shown in Fig. 2c. The method is based on a physiologic model of arterial wave reflection. This tube-load model is described in detail elsewhere^21^. Briefly, the tube accounts for the inertance [L] and compliance [C] of the large artery segment between the ascending aorta and brachial artery and thus offers constant characteristic impedance [Z_c_ = √(L/C)] and permits waves to travel along it with constant pulse transit time [T_d_ = √(LC)]. The load accounts for the small artery resistance [R]. Waves traveling in the forward direction along the tube are reflected in the opposite direction at the terminal load with a constant reflection coefficient [Γ = (R − Z_c_)/(R + Z_c_)] so as to mimic the well-known amplification of brachial pulse pressure (PP) relative to central PP.

According to this model, the transfer function relating the brachial BP waveform [p_b_(t)] (i.e., BP at the tube end) to the central BP waveform [p_c_(t)] (i.e., BP at the tube entrance) may be defined in terms of two parameters, T_d_ and Γ (see transfer function equation in the time-domain in Fig. 2c). As explained elsewhere^21^, this transfer function is often insensitive to Γ. Hence, this parameter could be fixed to a nominal value without significantly compromising accuracy. On the other hand, T_d_ is a vital transfer function parameter. In particular, application of the transfer function predicts high PP amplification (ratio of brachial PP to central PP) when T_d_ is large and low PP amplification when T_d_ is small. It is well known that pulse transit time is strongly related to MP and other variables. Hence, T_d_ may be reasonably predicted from readily available measurements and thereby adapt to some extent to the inter-subject and temporal variations in PP amplification. The nominal value for Γ and the prediction equation for T_d_ were established using a training dataset (see Fig. 2c). The T_d_ prediction equation capitalizes on the inverse relationship between pulse transit time and MP, which is due to slack collagen fibers in the arterial wall and aging^24^. Note that since this equation is fixed for all patients, the transfer function is not patient-specific. However, the equation allows for a transfer function that can vary (as opposed to the conventional GTF) and is simple enough that it may generally hold.

So, first, MP, computed as the time average of the brachial BP-like waveform over its foot-to-foot interval, is used to predict T_d_. Then, the fully defined VTF is applied in the time-domain to the entire brachial BP waveform to compute the central BP waveform.

### Patient Data

#### Experimental Procedures

To investigate the physiologic method for measuring the central BP waveform, patients admitted for diagnostic cardiac catheterization at Taipei Veterans General Hospital (Taiwan) were studied. The study procedures were approved by the hospital’s IRB and conformed to the principles of the Declaration of Helsinki. Written, informed consent was obtained from each patient.

The data collection procedures are described in detail elsewhere^9,25^. Briefly, all patients had inter-arm cuff BP differences of no more than 3 mmHg. A high-fidelity catheter with one or two micromanometers (SPC-320 or SSD-1059, Millar Instruments, USA) was positioned in the ascending aorta and brachial artery to sequentially or simultaneously measure gold standard reference central and brachial BP waveforms. An appropriately sized, inflatable cuff of a special office device (WatchBP Office, Microlife AG, Switzerland or VP-1000, Omron Colin, Japan) was placed properly over the other brachial artery to measure the cuff pressure waveform via conventional deflation, a PVP waveform via maintenance of the cuff pressure at 60 mmHg (“sub-diastolic PVP” waveform) for 30 sec, and the brachial BP levels estimated by the device. All of these cuff measurements were obtained during each sequential BP waveform measurement or the simultaneous BP waveform measurement under baseline and/or sublingual nitroglycerin conditions. Repeated cuff measurements were made per condition for the Microlife device.

#### Data Selection

All sets of cuff pressure and BP measurements were screened for possible exclusion from subsequent analysis. The exclusion criteria for a measurement set were: (a) substantial artifact due to motion or otherwise in at least one waveform as determined by visual inspection; (b) MP difference in brachial and central BP waveforms, which are sequentially (as opposed to simultaneously) measured, exceeding 5 mmHg; or (c) BP waveforms, which are sequentially measured, during the transient nitroglycerin condition. The latter two criteria ensured that the central and brachial BP waveforms were indicative of the same physiologic state. About 120 patients were included for study, and a total of 209 measurement sets from 87 patients remained for analysis. The measurement sets from 36 of the patients were previously used to develop the patient-specific method for estimating brachial BP levels, so these data constituted the training dataset. The measurement sets from the other 51 patients formed the testing dataset. Table 1 shows the measurement and patient characteristics for the datasets. Note that the testing dataset included Omron and Microlife cohorts.

**Table 1:** Measurement and Patient Characteristics.

		Training	Testing		
			Cohort 1		Cohort 2
Measurements					
	Device	Microlife	Omron		Microlife
	Device measurements	deflation cuff pressure waveform + office brachial BP levels + sub-diastolic PVP waveform			
	Reference	invasive brachial and central BP waveforms			
	# of subjects	36	43		8
	# of baseline measurements	36	38		8
	# of nitroglycerin measurements	36	13		8
	# of repeated measurements	70	0		10
	Total # of measurements	142	51		26
Patients					
	Type	Cardiac catheterization			
	Age [years]	64.9 ± 12.6	57.1 ± 13.9		71.2 ± 12.7
	Weight [kg]	75.7 ± 13.1	69.7 ± 12.1		69.3 ± 14.9
	Height [cm]	161.8 ± 8.2	163.5 ± 8.8		161.2 ± 10.5
	Waist circumference [cm]	90.4 ± 12.5	92.6 ± 11.5		94.5 ± 11.0
	Men [%]	75.7	75.0		75.0
	Smoking [%]	18.9	20.5		25.0
	Hypertension [%]	59.5	56.8		87.5
	Type 2 Diabetes mellitus [%]	29.7	31.8		50.0
	Dyslipidemia [%]	37.8	40.9		37.5
	Coronary artery disease [%]	59.5	56.8		62.5
	Chronic renal failure [%]	2.7	2.3		12.5
	α-Blockers [%]	13.5	11.4		25.0
	β-Blockers [%]	43.2	38.6		62.5
	Calcium channel blockers [%]	48.6	40.9		25.0
	Diuretics [%]	18.9	20.5		37.5
	Antiplatelet agents [%]	86.5	70.5		87.5

### Data analysis

The training dataset was analyzed to develop the sub-methods of the physiologic method. The patient-specific method was rigorously developed as described elsewhere^19^, whereas simple, but sub-optimal, approaches were applied here to develop the ensemble averaging and VTF methods. For comparison, the training dataset was also used to build the conventional method of Fig. 1a.

To develop the ensemble averaging method, the variable amplitude cuff pressure oscillation waveforms and sub-diastolic PVP waveforms were analyzed. In particular, the user-selected variables of the method were established so that the RMSE of the deflation PVP waveform extracted from the variable amplitude waveform with respect to the corresponding sub-diastolic PVP waveform (formed by conventional ensemble averaging and amplitude normalization for the average waveform beat but not the individual waveform beats) was <0.1.

To develop the VTF method, the sub-diastolic PVP waveforms, simultaneously measured central BP waveforms, and invasive brachial BP waveforms were analyzed. The sub-diastolic PVP waveforms were first calibrated to invasive brachial DP and SP so as to avoid over-fitting the transfer function to random calibration error. For each pair of brachial BP-like and central BP waveforms, Γ and T_d_ were estimated by least squares fitting of the model predicted central BP waveform (see Fig. 2 c) to the measured central BP waveform. The value of Γ was then set to the average of the Γ estimates. A T_d_ prediction equation was created using the T_d_ estimates as the dependent variable and various measurements as the independent variables. The investigated independent variables included the invasive brachial BP levels (to likewise prevent overfitting of the equation), the brachial artery compliance parameter estimates of the patient-specific method, pulse rate, and patient anthropomorphic data such as age, height, and arm circumference. Multivariate linear regression was employed, and the utility of the independent variables was assessed using a step-wise approach. MP was concluded to be the only independent variable in the final prediction equation (see Fig. 2 c). The correlation coefficient between the predicted and measured T_d_ was almost 0.6. PTT limits were thereafter added to the T_d_ prediction equation to protect against gross MP estimation error (see Fig. 2c).

To develop the conventional method, various possible implementations were explored, and the best implementation was selected. In particular, the GTF was defined in terms of the tube-load model of Fig. 2c or an autoregressive exogenous input model^21^. To set the model parameters of the GTF, the sub-diastolic PVP waveforms were calibrated to invasive brachial SP and DP, invasive brachial MP and DP, the brachial SP and DP obtained with the office device, or the brachial MP and DP obtained with this device. The selected GTF was based on the tube-load model with parameters set to the averages of the aforementioned Γ and T_d_ estimates. This parameter setting is justifiable, because the sub-diastolic (rather than deflation) PVP waveform was used and calibrated to invasive (instead of patient-specific) brachial SP and DP in the development of the VTF method. Note that this GTF must be evaluated as applied to the PVP waveform calibrated with the brachial SP and DP obtained with the office devices rather than an invasive catheter system. However, the office devices were likely developed based on reference auscultation BP measurements, which systematically underestimate invasive brachial SP and overestimate invasive brachial DP^26^. Hence, prior to the PVP waveform calibration, the office brachial BP levels were corrected in terms of their bias error (see below). Such a bias correction allowed the GTF to serve its intended purpose of reducing PP amplification, significantly improved the central BP measurement accuracy of the conventional method, and could easily be implemented in practice. Finally and importantly, the other implementations of the conventional method did not improve the central BP waveform accuracy in the training or even testing datasets (results not shown).

The testing dataset was then analyzed to assess and compare the accuracy of the developed methods. The physiologic method as well as the physiologic method with the VTF replaced by the GTF were applied to the standard cuff pressure waveforms, whereas the conventional method was applied to the additional, sub-diastolic PVP waveforms calibrated to the brachial SP and DP estimated by the office device from the standard cuff pressure waveforms. For reasons mentioned above, prior to PVP waveform calibration, the office brachial BP levels were corrected so that their bias errors were the same as those of the patient-specific method for each of the two patient cohorts. The errors between the resulting brachial and central SP, MP, DP, and PP measurements and the gold standard reference BP levels were quantified via the conventional bias error (i.e., mean of the errors) [μ] and precision error (i.e., standard deviation of the errors) [σ]. The bias and precision errors for the lower, middle, and upper tertile PP amplification subgroups were also computed to investigate the added value of the VTF method.

### Statistical Methods

The bias and precision errors of two methods were compared via paired t-tests and Pitman-Morgan tests^27^, respectively. To generously account for multiple comparisons, a p ≤ 0.01 was considered significant.

### Data availability

The data analyzed in this study may be available from H.M.C., S.H.S., and C.H.C. via reasonable requests made to the corresponding author.

## Results

The training dataset was needed to develop the methods for investigation. However, the results from this dataset carry little meaning and did not offer additional insight. Hence, only the testing dataset results are provided.

### Patient BP Levels

Table 2 shows the average ± SD and range of reference brachial and central SP, MP, DP, and PP as well as PP amplification (ratio of brachial PP to central PP). All of the BP parameters varied widely. Most notably, central SP and PP ranged over 105 and 82 mmHg, respectively.

**Table 2:** Reference Blood Pressure (BP) Parameters in the Testing Dataset.

	SP [mmHg]	MP [mmHg]	DP [mmHg]	PP [mmHg]	PP Amplification [unitless]
Brachial	134 ± 21 (99–192)	96 ± 13 (72–129)	71 ± 11 (43–101)	63 ± 19 (33–113)	1.2 ± 0.15 (0.99–1.7)
Central	125 ± 23 (85–190)	95 ± 13 (69–128)	73 ± 10 (47–101)	53 ± 20 (26–108)	

Values are average ± SD (minimum – maximum). SP, MP, and DP are systolic, mean, and diastolic BP, respectively; PP, pulse pressure; and PP amplification, ratio of brachial PP to central PP.

### Brachial BP Measurement Accuracy

Table 3 shows the brachial SP, DP, and PP bias and precision errors (average ± SE) of the patient-specific method and the office devices. The patient-specific method yielded significantly lower precision errors than the office devices and thereby afforded superior calibration. As expected, the patient-specific method also produced significantly lower bias errors. However, the office device bias errors could be corrected in practice (by e.g., adding and subtracting constant values from brachial SP and DP). Hence, in this study, the BP levels of the office devices were adjusted to make their bias errors equal to those of the patient-specific method.

**Table 3:** Brachial BP Bias and Precision Errors (average ± SE) in the Testing Dataset.

Method	Brachial SP [mmHg]		Brachial DP [mmHg]		Brachial PP [mmHg]	
	μ	σ	μ	σ	μ	σ
Omron	−5.7 ± 1.2	10.7 ± 0.9	2.7 ± 1.1	9.5 ± 0.8	−8.4 ± 1.5	12.9 ± 1.1
Patient-specific	0.7* ± 1.0	8.8* ± 0.7	3.5 ± 0.8	7.3* ± 0.6	−2.8* ± 1.1	9.4* ± 0.8
Microlife	−4.5 ± 1.2	10.6 ± 0.9	4.4 ± 0.6	5.4 ± 0.4	−8.9 ± 1.5	13.2 ± 1.1
Patient-specific	−3.4 ± 0.9	7.5* ± 0.6	−1.1* ± 0.7	5.8 ± 0.5	−2.3* ± 1.1	10.0* ± 0.8

*p ≤ 0.01 compared to corresponding office device via paired t-test for bias error (μ) and Pitman-Morgan test for precision error (σ).

### Central BP Measurement Accuracy

**Figure 3:**
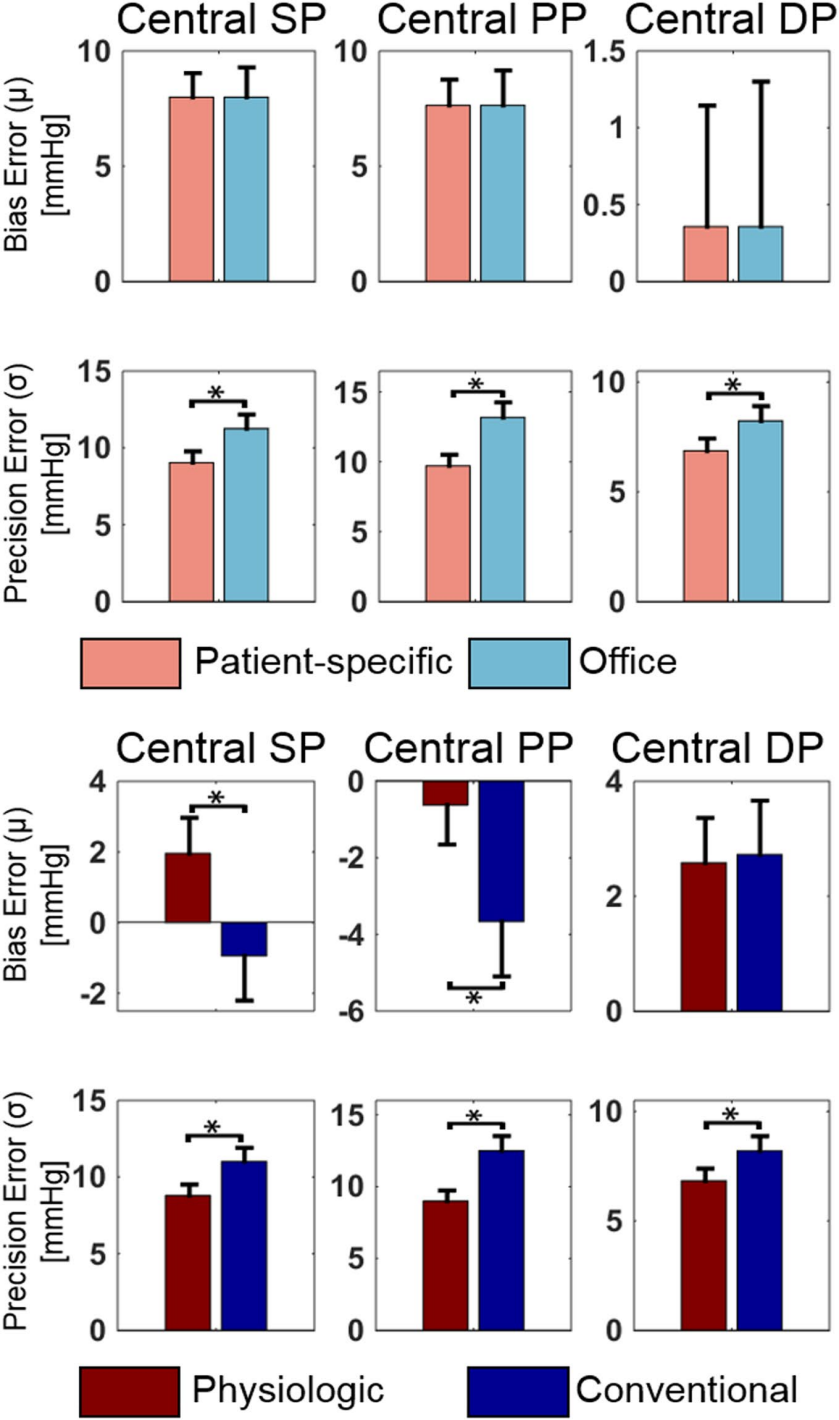
Central SP, pulse pressure (PP), and DP bias errors (μ) and precision errors (σ) (average ± SE) of the patient-specific method (Fig. 2a) versus the office device (a population average method) and the physiologic method (Fig. 1b) versus the conventional method (Fig. 1a) in the testing dataset. Note that the patient-specific method and office device measure brachial BP levels, so the upper plots represent errors between brachial BP levels and central BP levels (i.e., starting point errors prior to applying the transfer function). *p ≤ 0.01 compared to corresponding method via paired t-test for μ and Pitman-Morgan test for σ.

**Figure 4:**
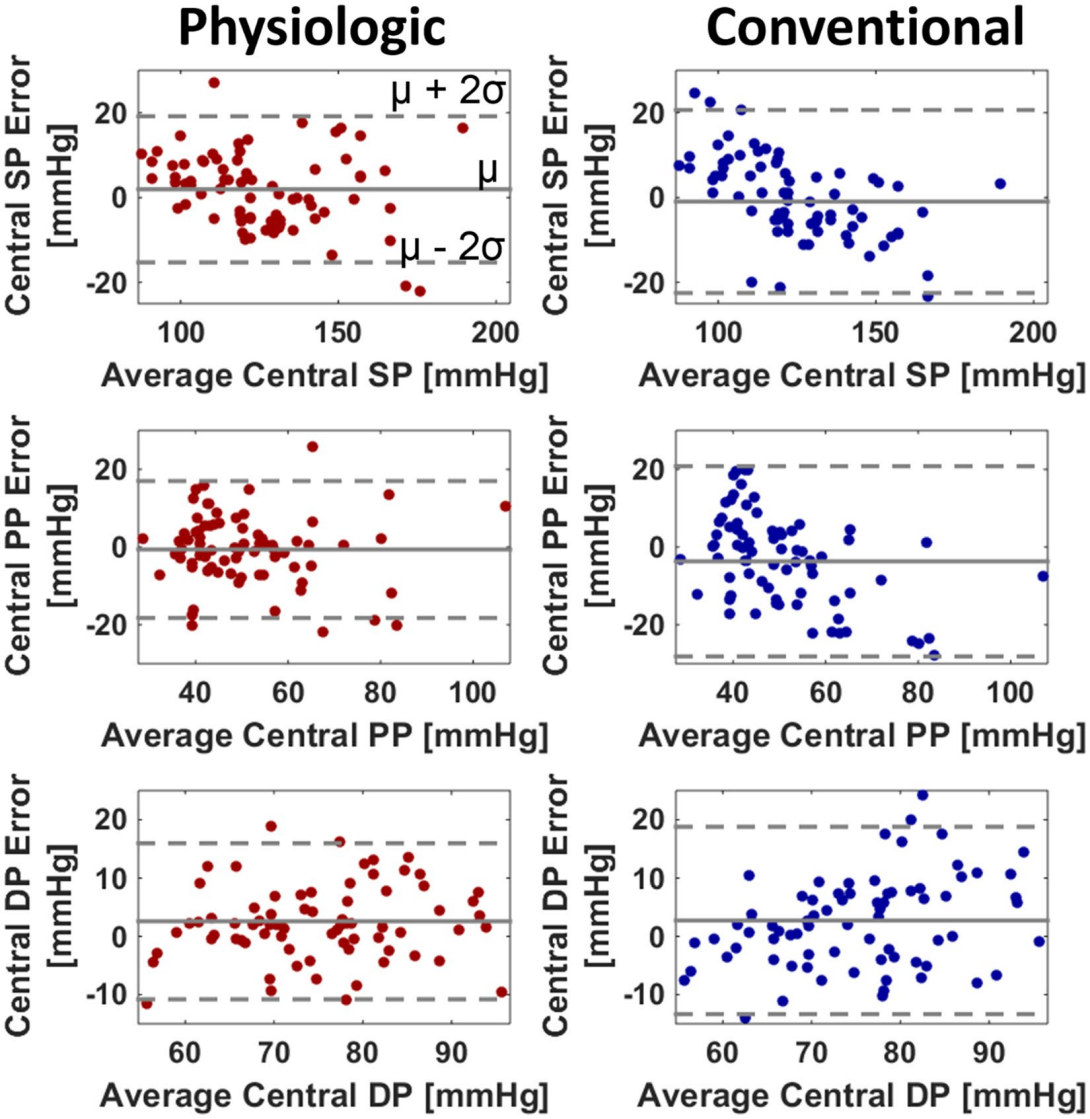
Bland-Altman plots of the central SP, PP, and DP errors of the physiologic method and conventional method in the testing dataset.

Fig. 3 shows the central SP, PP, and DP bias and precision errors (average ± SE) of the patient-specific method versus the office device (top) and of the physiologic method versus the conventional method (bottom) aggregated over both cohorts. (The precision errors for each cohort were along the lines of Table 3). The central BP errors of the patient-specific method and office device (i.e., the errors between the brachial BP levels measured by these methods and the reference central BP levels) represent the “starting point” errors prior to applying the transfer function. As expected, the central SP and PP bias errors were large and positive. While the two methods yielded the same bias errors due to the bias correction, the patient-specific method produced significantly lower precision errors. Comparing the precision errors to those in Table 3, it can be inferred that the main source of these errors is the calibration error rather than PP amplification variability. Application of the transfer function reduced the central SP and PP bias errors greatly but not the corresponding precision errors (compare top to bottom). The physiologic method afforded central BP bias errors of −0.6 to 2.6 mmHg and precision errors of 6.8 to 9.0 mmHg. These errors were significantly lower than those of the conventional method by 22% in terms of average RMSE. This error reduction was mainly due to improved PVP waveform calibration. Fig. 4 shows Bland-Altman plots of the errors of the two featured methods for comparison.

**Figure 5:**
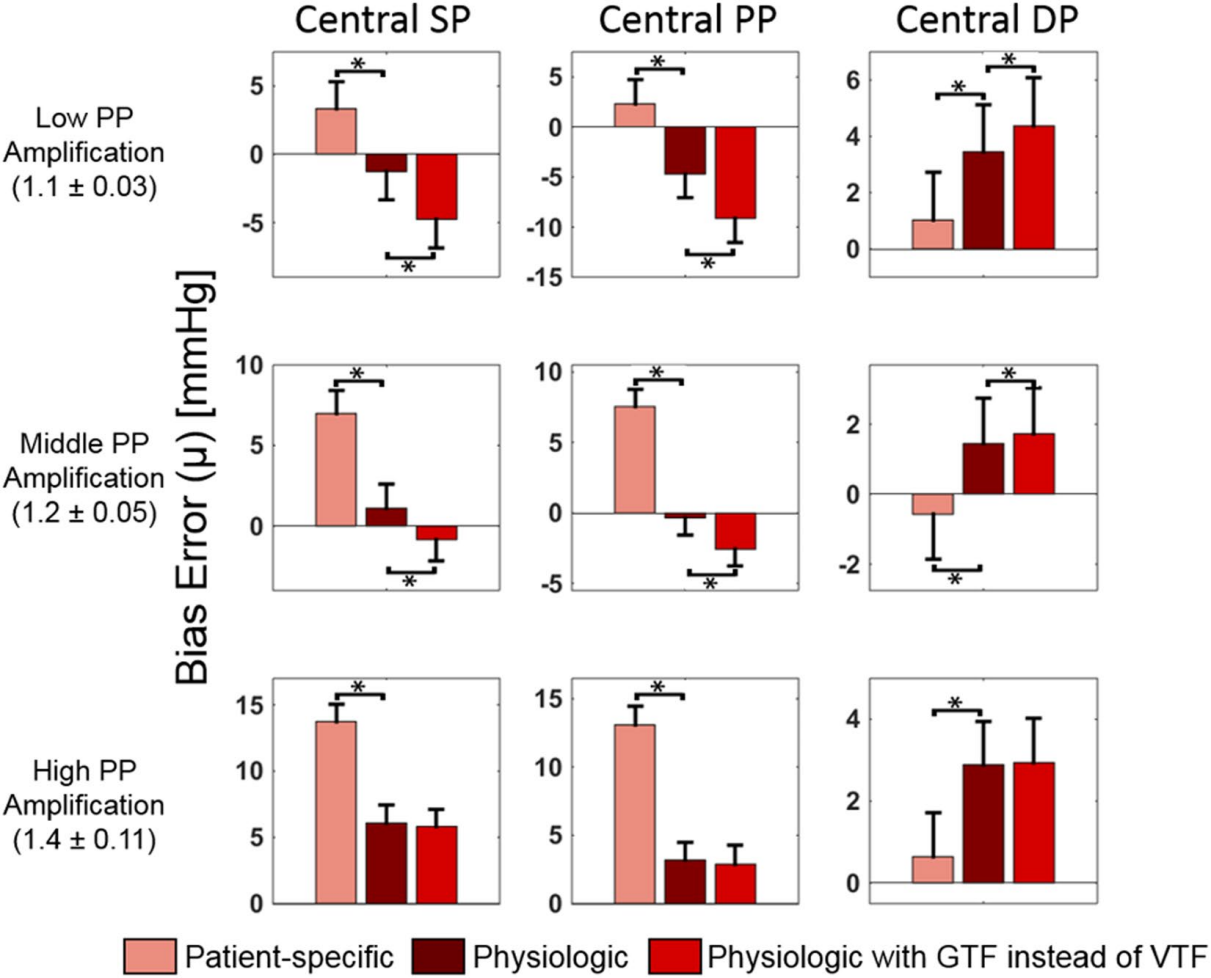
Central SP, PP, and DP bias errors (average ± SE) of the patient-specific method versus the physiologic method versus the physiologic method with VTF replaced by GTF for different PP amplification (ratio of reference brachial PP to central PP) subgroups in the testing dataset. Again note that the patient-specific method measures brachial BP levels. *p ≤ 0.01 between physiologic method and other method via paired t-test. The precision errors of the methods were similar for the subgroups.

Fig. 5 shows the central SP, PP, and DP bias errors (average ± SE) of the patient-specific method versus the physiologic method versus the physiologic method with the VTF replaced by the GTF for the low, middle, and high PP amplification subgroups. (The precision errors were similar amongst the methods.) The purpose of this figure is to reveal the value of the VTF method. As expected, the patient-specific method (which again measures brachial rather than central BP levels) yielded central SP and PP bias errors that were large and positive when PP amplification was high and that decreased appreciably with PP amplification. As also expected, the GTF significantly decreased the central SP and PP bias errors by mitigating the overestimation of these BP levels when PP amplification was higher but substantially increased the errors by underestimating central SP and PP when PP amplification was low. The VTF provided significantly lower central SP and PP bias errors over the whole PP amplification range by decreasing the pulse transit time parameter of the tube-load model transfer function with increasing MP. However, it was not always superior. While the VTF reduced or maintained the central SP and PP bias errors of the GTF, its added value overall was not large due to the higher precision errors of both methods (see Fig. 3). On the other hand, the patient-specific method yielded significantly lower central DP bias errors. Hence, the patient-specific DP could instead be used to improve central DP accuracy to a mild extent.

### Accuracy of the Intermediate Variables for Measuring Central BP

The ensemble averaging method yielded a RMSE of the deflation PVP waveform with respect to the corresponding sub-diastolic PVP waveform of 0.07±0.03. The time average of the deflation PVP waveform calibrated with patient-specific brachial SP and DP yielded MP bias and precision errors of 4.3 and 7.8 mmHg. Finally, the T_d_ prediction equation produced a correlation coefficient between predicted and measured T_d_ of 0.5.

## Discussion

We proposed a “physiologic method” to monitor the central BP waveform via a standard automatic arm cuff (see Fig. 1b). The method applies three sub-methods in succession as follows. First, a patient-specific method that we recently introduced^19^ is employed to estimate brachial BP levels from a cuff pressure waveform obtained during conventional deflation by leveraging a physiologic model and optimization (see Fig. 2a). This method can yield more accurate brachial BP levels than current population average methods, as we showed previously^20^ and herein (see Table 3), and may thus reduce the major calibration error of most current tonometric and oscillometric devices for non-invasive monitoring of central BP^2,14,15^. Then, an ensemble averaging/calibration method is applied to the same cuff pressure waveform so as to extract a “deflation PVP” waveform and scale it to patient-specific brachial SP and DP (see Fig. 2 b). This simple, yet physiology-based, method may eliminate the need for the additional step performed by all available oscillometric devices in which the cuff is maintained at a constant pressure to measure the PVP waveform, which is then calibrated to the population average brachial BP levels (see Fig. 1 a). Finally, a VTF method is employed to convert the brachial BP-like waveform to the central BP waveform. The method defines the transfer function in terms of the pulse transit time (T_d_) and wave reflection coefficient (Γ) parameters of a physiologic model (see Fig. 2 c). The reflection coefficient is set to a nominal value, as the transfer function is often insensitive to this parameter, while the pulse transit time, which has significant impact on the extent to which the transfer function reduces PP amplification, is predicted based on its well-known inverse relationship with MP (see Fig. 2 c). This simple, physiologic modeling method may thus adapt the transfer function to BP-induced changes in arterial stiffness unlike the GTF, which is utilized by most of the current tonometric and oscillometric devices (see Fig. 1a). In this way, central BP could be measured – for the first time – both reliably and in the exact same way as traditional brachial cuff BP.

We developed and evaluated the physiologic method for measuring the central BP waveform using data from cardiac catheterization patients (see Table 1). These data included the cuff pressure waveform obtained during conventional deflation, the brachial BP levels estimated from this waveform by popular office devices, a “sub-diastolic PVP” waveform obtained during constant inflation at 60 mmHg, and gold standard invasive reference central and brachial BP waveforms. In the testing dataset, the reference BP parameters varied widely (e.g., central SP ranged from 85 to 190 mmHg) mainly due to differing degrees of patient arterial stiffness (see Table 2). The precision errors between the brachial SP and PP computed by the office device and reference central SP and PP were 11.3 and 13.2 mmHg, respectively (see Fig. 3). These high “starting point” errors together with the wide BP parameter range underscored the challenge presented by the testing dataset.

The physiologic method yielded central SP, DP, and PP bias errors within 2.6 mmHg in magnitude and precision errors within 9 mmHg (see Figs 3 and 4). These errors nearly satisfied the AAMI limits of 5 and 8 mmHg, though an AAMI data collection protocol was not employed.

We also compared the physiologic method to the conventional oscillometric method in which a GTF is applied to a sub-diastolic PVP waveform calibrated with office brachial BP levels to derive the central BP waveform (see Fig. 1a) ^4,5,8^. Since the GTF was built using invasive brachial SP and DP, the office devices were likely built using auscultation rather than invasive BP as the reference, and there is systematic error between the two reference methods^26^, the bias errors of the office brachial BP levels (see Table 3) were first corrected to be the same as the patient-specific method. A GTF defined by the tube-load model in Fig. 2c, but with average values for both parameters, was then applied. Note that the bias correction was necessary to improve the accuracy of the conventional method and could be performed in practice. Importantly, other possible implementations of the conventional method (i.e., different PVP calibration procedures with and without bias correction and different GTFs) did not measure central BP levels more accurately. Hence, the employed conventional method may represent the best possible implementation.

Compared to the conventional method, the physiologic method produced significantly lower central SP, DP, and PP errors (see Figs 3 and 4). Overall, the physiologic method yielded a 22% error reduction. The improved calibration afforded by the patient-specific method for measuring brachial BP levels was the main contributor to the reduction (see Table 3 and Fig. 3). The transfer function adaptation to BP-induced arterial stiffness changes offered by the VTF method was a secondary contributor and was most helpful relative to the GTF method in patients with low PP amplification (see Fig. 5) where it was able to reduce the average central BP RMSE by 10%. The VTF method did not reduce the error compared to the GTF method in patients with high PP amplification, as the Td prediction via MP actually underestimated Td on average. Hence, despite being imperfect, the simple VTF method was still good enough to yield an improvement in central BP measurement accuracy in patients not used in its development. Further, the deflation PVP waveforms produced by the ensemble averaging method were similar enough to the sub-diastolic PVP waveforms that they hardly impacted the central BP errors (results not shown).

Other methods for central BP monitoring via an automatic arm cuff are available that instead obtain a supra-systolic PVP waveform and/or compute central BP from a calibrated PVP waveform without using a GTF. One method applies a transfer function based on the tube-load model in Fig. 2c to a calibrated, supra-systolic PVP waveform to derive the central BP waveform^12^. The interesting idea is that, when the brachial artery is occluded by the supra-systolic cuff inflation, the forward and backward waves will be equal in magnitude^28^. In this way, Γ is correctly determined as unity. However, the transfer function is often insensitive to Γ^21^, as we have mentioned, and whether the more important T_d_ can be well determined from the proposed time delay between systolic PVP peaks or not is less certain. Further, the main source of error is the calibration rather than the transfer function, and the supra-systolic PVP waveform is small and thus susceptible to noise. Another method, which some of us developed, applies a multiple regression equation to several features of a calibrated, sub-diastolic PVP waveform of about 30 sec in duration to predict central SP and PP^9–11^. This equation can yield significantly smaller central PP errors than a GTF by effectively reducing the calibration error^11^. The reported precision errors of the method are also lower than those herein for the physiologic method^10^, but the patient data for evaluation were not the same. The error differences could also be explained by the fact that the central BP waveforms derived by the physiologic method were obtained from single cuff deflation measurements, whereas the central BP levels predicted by the regression method represented the average of two cuff deflation measurements. Such averaging can reduce the precision error by a factor of up to 1/√2. In any case, future comparisons of the physiologic method with other methods should be performed using the same data and analyses to obtain a conclusive assessment of their relative accuracy.

Even if other methods prove more accurate than the physiologic method in head-to-head comparisons, the difference would presumably have to be large enough to justify their additional cuff inflation. Automatic arm cuffs are already cumbersome enough to use^24^. Requiring a prolonged sub-diastolic PVP waveform measurement, which could approximately double the measurement time, or a supra-systolic PVP waveform measurement, which is uncomfortable to the subject, may reduce patient compliance for using the device. Conversely, a method for measuring central BP with an acceptable level of error, but without changing the traditional measurement procedure, could increase the adoption of central BP.

This study has limitations. One limitation is that the data were not homogeneous (see Table 1). For example, two office devices (Microlife and Omron) were employed. Such heterogeneity could have added variability to our results. On the other hand, any variability introduced by the use of two devices may not have been substantial (see Table 3). Another limitation pertains to the VTF method. This transfer function neither accounts for differences in the shapes of brachial PVP and BP waveforms due to viscoelastic effects^29^ nor is patient-specific (i.e., truly adaptive). That said, a superior transfer function method would not have made a major difference here, as the calibration error dominated. Adaptive transfer functions, such as those proposed by some of us^21,22^, may offer greater value when calibration error is not a factor such as when invasive peripheral BP waveforms are available or when calibrated radial artery tonometry waveforms are converted to likewise calibrated carotid artery tonometry waveforms.

In conclusion, PP and SP are amplified in the brachial artery relative to the central aorta. So, it is central BP that truly affects cardiac performance. Moreover, central BP rather than brachial BP is a major determinant of the degenerative changes that occur in aging and hypertension^30^. Hence, central BP could provide greater clinical value than brachial BP. While several studies have demonstrated the added value of central BP^1^, the extent of the difference may be considered unsatisfying. One possible explanation is that non-invasive central BP measurements suffer from substantial error due to the error introduced by the calibration step, which can be similar in magnitude to the difference between central and brachial BP levels. Another explanation is that the tonometric devices that have long been available for non-invasive central BP monitoring are not convenient enough for central BP to be studied broadly. We introduced a physiologic method to both mitigate the calibration error and obtain central BP measurements in the exact same way as traditional automatic cuff BP measurements. We showed that this method can yield central BP measurements that agree with gold standard reference measurements to a significantly greater degree than some current non-invasive devices. Future investigations may be worthwhile to confirm the accuracy of the new method, especially in a real-time device, investigate the method during improper cuff usage, and apply it broadly to determine the full clinical potential of central BP.
